# Surgical versus non-surgical treatment of intra-articular comminuted distal radius fractures (AO 23-C2/C3) is associated with better patient-reported outcomes: an instrumental variable analysis using a national Swedish cohort

**DOI:** 10.1186/s12891-026-09900-z

**Published:** 2026-05-04

**Authors:** Albert Christersson, Jonas Höijer, Michael Möller, Karl Michaëlsson

**Affiliations:** 1 Orthopaedics, Department of Surgical Sciences, Uppsala University, Uppsala, SE-75185 Sweden; 2 Department of Surgical Sciences, Medical Epidemiology, Uppsala University, Uppsala, Sweden; 3 Orthopaedics, Institute of Clinical Science, Sahlgrenska Academy, University of Gothenburg, Gothenburg, Sweden

**Keywords:** Distal radius fracture, Surgical treatment, Non-surgical treatment, AO-23 A, AO-23 C, Instrumental analysis, Swedish fracture register

## Abstract

**Background:**

The surgical rate for distal radius fractures is steadily rising despite limited evidence of its benefits over non-surgical treatment. Using a natural experimental approach, we aimed to compare patient-reported outcomes following surgical versus non-surgical treatment of distal radius fractures.

**Methods:**

Registered in the Swedish Fracture Register by 36 Swedish hospitals from 2013 to 2018, we included a cohort of 13,356 fractures on 13,031 patients aged 18 years or older with distal radius fractures Arbeitsgemeinschaft fur Osteosynthesefragen (AO) 23-A2.1–2, A3, and C1-C3. The observational study utilized differences in the frequency of surgical treatment across hospitals as a source of random treatment assignment and a natural experiment. We assumed that all hospitals encountered a similar range of fractures each year. Therefore, the annual frequency of surgery per hospital was used as a proxy for randomization between surgical and nonsurgical treatment, regardless of each patient's actual treatment. The outcome was the individual Patient Reported Outcome Measures (PROM) at 1 year, with the Arm and Hand Function Index from the Short Musculoskeletal Function Assessment (SMFA) as the primary measure.

**Results:**

The surgical rate per hospital year ranged from 7 to 66%. Surgical treatment was associated with lower Arm and Hand Function Index scores in comminuted intraarticular fractures of type C2 (11.9 units, *p =* 0.004) and type C3 (19.4 units, *p =* 0.029). There was a tendency for a positive association with surgical treatment in dorsally angulated extraarticular fractures (23A2.2), but the difference of 5.1 units (*p =* 0.079) was below the Minimal Clinically Important Difference (MCID). In other extra-articular fractures (23-A2.1 and 23-A3) and simple intra-articular fractures (23-C1), the benefits of surgical treatment were small and also not statistically significant. Several sensitivity analyses were conducted to test the study design, and all supported the primary results.

**Conclusions:**

In this comparison of surgical and non-surgical treatment for distal radius fractures across hospitals with varying surgical rates, comminuted intra-articular distal radius fractures (AO 23-C2/C3) treated surgically were associated with better one-year patient-reported outcomes than those treated non-surgically.

**Supplementary Information:**

The online version contains supplementary material available at 10.1186/s12891-026-09900-z.

## Introduction

Distal radius fractures (DRF) are the most common orthopedic injury, occurring two to three times more often than hip fractures. In adults, they can serve as a prognostic marker for a future hip fracture, which is considered the most severe fragility fracture [1, 2].

Non-surgical treatment is the most common treatment for DRF, but after the volar plate was introduced as a treatment option in 2005–2006, the surgical rate has steadily increased [3]. Nonetheless, although surgical versus non-surgical treatment of displaced DRFs has been examined in several randomized controlled trials (RCTs), the indication for surgical treatment remains unclear. The ambiguities regarding the benefits of surgery for distal radius fractures are further underscored by two recent meta-analyses based on almost the same RCTs comparing different surgical treatments with nonoperative treatment. One meta-analysis [4] based on 14 studies concluded that surgical treatment has a positive effect on unstable DRFs while another [5], based on 12 RCTs, found no clinically meaningful benefit with surgical treatment. This conflicting evidence reveals a lack of clear understanding about whether surgical treatment is preferable and which DRF type would preferentially be treated by surgery.

The goal of our national register-based cohort study was to estimate the effectiveness of implementing surgical treatment for DRF in routine clinical care by examining differences in the timing and rate of surgery adoption across Swedish hospitals. Since surgery was gradually introduced and some hospitals adopted it earlier than others, a patient's chance of receiving surgery depended on when and where they were hospitalized for a DRF. By leveraging this gradual implementation and accounting for hospital-specific variations in surgical treatment rates as a natural experiment [6], we could approximate random variation in treatment assignment to assess how increased use of surgery for DRF impacted 12-month patient-reported outcomes in Swedish routine clinical practice.

## Methods

We combined a cohort design based on the national Swedish Fracture Register (SFR) with an instrumental variable analysis [6] to compare surgical and non-surgical treatment in patients with a closed distal radius fracture (ICD S52.50), aged ≥ 18 y, registered in the SFR with injury date between January 1 st, 2013, and December 31 st, 2018.

**Fig. 1:**
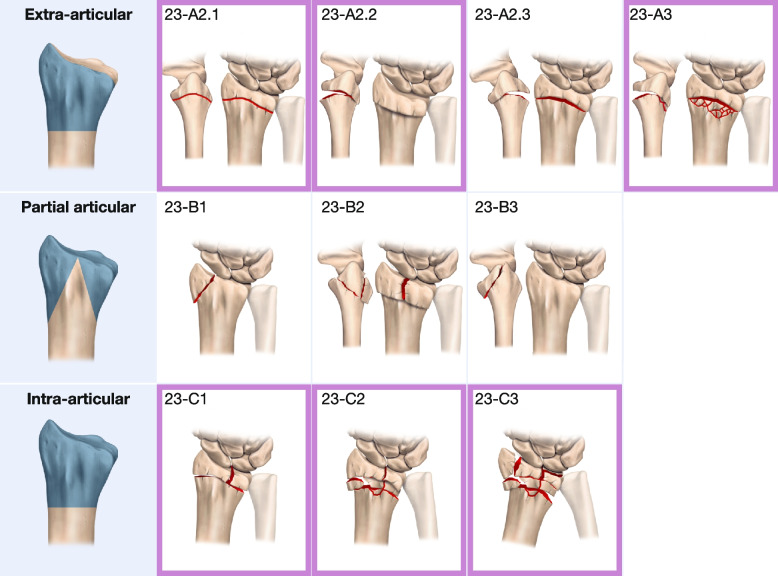
AO-classification of distal radius fractures. AO-23 classes included in the study are marked with purple frames. Illustration made by Pontus Art Production

**Fig. 2:**
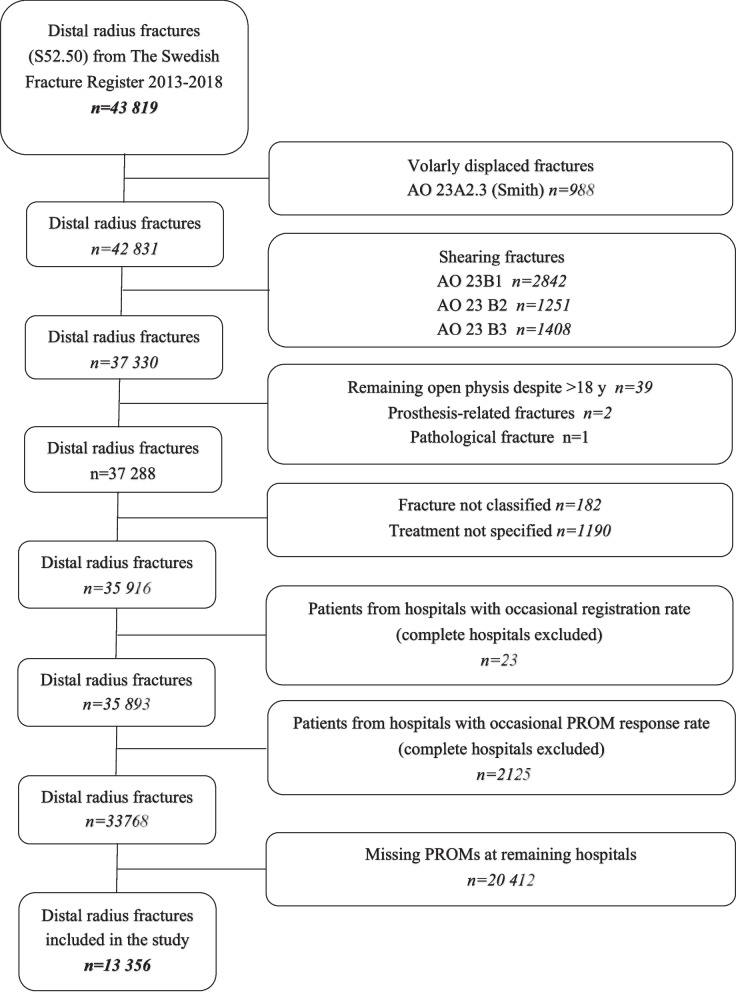
Flow chart of patients with distal radius fractures in 2013–2018 derived from the Swedish fracture register

Volarly displaced fractures (23-A2.3), intra-articular shearing fractures (23-B1-B3) (Fig. 1), fractures with remaining open physis, prosthetic-related fractures, pathological fractures, and fractures without a specified fracture classification or treatment were excluded. Hospitals with only occasional registration or low response rates due to insufficient routines were also excluded (Fig. 2).

Compared with the out- and in-patient National Patient Register, completeness of distal radius fracture registrations in SFR ranges from 31 to 96% (mean approximately 70%) among the 47 departments registering fractures in 2020 [7]. Before exclusions, the data comprised 43,819 fractures from 45 hospitals. A total of 33,768 fractures in 33,018 patients from 36 hospitals fulfilled the inclusion criteria. Of these, 13,356 fractures in 13,031 patients returned the PROM questionnaires, constituting the final analysis cohort (Fig. 2).

The SFR collects patient characteristics, energy level (high/low), trauma mechanism, AO classification, type of treatment, and Patient-Reported Outcome Measurements (PROMs, including EuroQol Group 5-dimension (EQ5D) [8] and the Short Musculoskeletal Function Assessment (SMFA) [9]. PROM questionnaires were sent to all patients registered in the Swedish Fracture Register within 2 weeks of the injury (PROM 0). To all patients who return PROM 0, the same questionnaire was sent 12 months after the injury (PROM 1).

The EQ5D has two parts: a five-dimension questionnaire (covering mobility, self-care, usual activities, pain/discomfort, and anxiety/depression), which generates the EQ5D index, and a visual analogue scale called the EQ5D VAS. Higher scores (0–1) indicate better well-being.

The SMFA includes two health indices. The first, the Dysfunction Index, has 34 items divided into four categories: daily activities, emotional status, arm/hand function, and mobility. The second, the Bother Index, consists of 12 items. Lower scores reflect better health status (0–100). The Arm and Hand Function Index, a subset of the SMFA Dysfunction Index, was used as the primary outcome in our study because it was the most relevant score for distal radius fractures [10]. The two EQ5D indices and the two SMFA indices were secondary outcomes. We also included the Mobility Index from SMFA as a secondary outcome for validation. Due to how the questions are formulated, the Arm Hand Function Index is regarded as a score for the upper extremity, and the Mobility Index is a score for the lower extremity. The minimal clinically important difference for the SMFA has been calculated to be 7 units [11], and for the Arm and Hand Function Index, 8 units [12]. For the EQ-5D Index, MCID in musculoskeletal disorders is 0.07–0.08 [13, 14], and for EQ-5D VAS 6,4 [14].

Our study assumes that all hospitals in Sweden see the same variety of patients and fractures. Therefore, the treatment outcome at each hospital depends solely on the type and quality of treatment provided. The first registration in the SFR was used as the treating hospital. Patients can be referred from elsewhere because the injury occurred while traveling outside their hometown or because a higher level of care was needed. Patients who received surgical treatment as their initial approach or underwent surgery after failing non-surgical treatment were categorized as “surgical treatment." Patients treated with a plaster cast, with or without prior fracture reduction, were classified as “non-surgical treatment." Radiographs were not included in the study.

The surgical rate varied both within hospitals and between hospitals over the entire period (2013–2018). Instead of using the average surgical rate per hospital as the instrument variable, we calculated each hospital's yearly surgical rate and used “Hospital year” (HY) as the comparison. This approach increased the contrast in surgical rates and reduced potential bias caused by hospitals with different skill levels in treating distal radius fractures.

We captured this variation in surgery assignment by using the hospital’s past annual surgery rate to estimate the likelihood that a patient with a DRF admitted to that hospital would undergo surgery. To evaluate the potential benefit of surgery on PROM, we employed two-stage least squares instrumental variable analysis to estimate a local average treatment effect [6]. This approach aimed to minimize bias that could distort the relationship between the exposure (surgery vs. non-surgery) and the outcome (PROM). Since many confounders operate at the individual level, we used the surgical rate per HY as an instrument for surgery, treating it as a natural experiment to obtain an unbiased estimate of surgery's effect on PROM. The models were adjusted for age, gender, and fracture class (AO 23-A2.1-A3 and C1-C3, Fig. 1). Statistical significance was set at *p <* 0.05. Additionally, we stratified the results by the AO classification for fracture type.

To assess the relevance of the instrument on the treatment, the Kleibergen–Paap F-statistic [15] was used in conjunction with the Stock-Yogo critical values [16], as well as partial R^2^. Since observations are dependent within hospital years, cluster-robust standard errors were used, which also account for potential heteroskedasticity.

We also conducted several sensitivity analyses to test the robustness of the results. First, we performed a sensitivity analysis excluding trauma level 1 hospitals to evaluate potential confounding from larger hospitals that might provide higher-quality treatment for DRF. Second, we repeated the same instrumental-variable analysis as in the main analysis but without including any covariates. Third, the association of interest was estimated using random-effects generalized least squares regression without an instrumental variable. Fourth, due to the skewness of the PROM distribution, a sensitivity analysis was performed in which PROM was dichotomized at different levels (from 3 to 45). For each cutoff, this binary variable served as the outcome in an instrumental variable logistic regression analysis using the R package “ivglm” [17]. Odds ratios comparing surgery versus non-surgery were calculated for each PROM cutoff. Fifth, we repeated the instrumental variable analysis after excluding patients with other injuries from one year before to one year after the index fracture, patients with another distal radius fracture during the study period, patients with bilateral fractures, patients treated surgically after initial non-surgical treatment or patients referred from one hospital to another due to consultation. Additionally, we performed an instrumental variable analysis comparing PROM 1 and PROM 0 (Delta PROM), rather than PROM 1 alone.

All analyses were performed using complete case analysis in Stata (v15.1; StataCorp, College Station, TX, USA) and R Statistical Software (v4.5.1; R Core Team 2025).

## Results

Among the 13,031 cohort patients with 13,356 distal radius fractures included in the analysis, the mean age at fracture was 64 years (range 18 to 99 years), and 83% were women. In the non-responder group, the mean age was 62 years, and 76% were women. The average number of patients per hospital was 382 (ranging from 8 to 1860), and the number of observation-years per hospital ranged from 1 to 6 (median 4.0). On average, each hospital treated 95 patients annually.

**Fig. 3:**
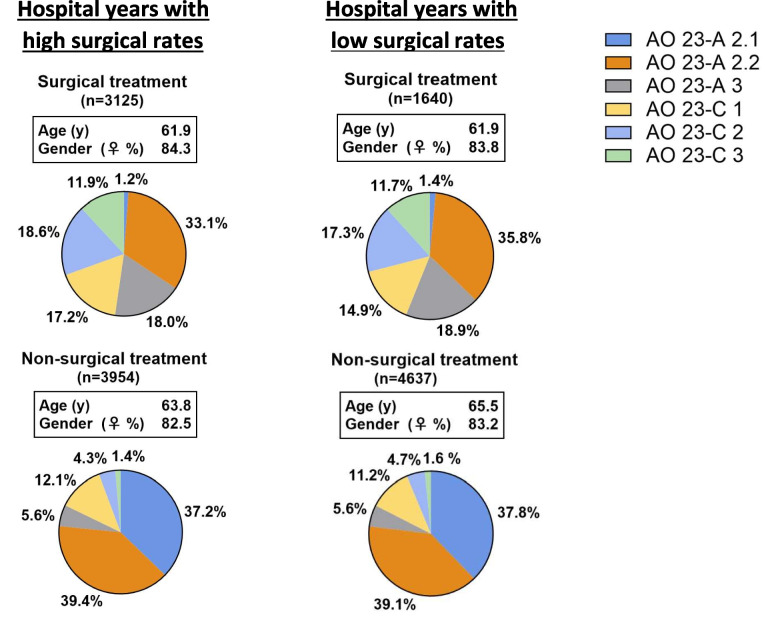
Distribution of AO-23 fracture classes, sex, and age in the final study cohort. Hospital years have been dichotomised to lower (< 31%) or higher (> 31%) than the average surgical rate

There was an even distribution of fracture classes across hospitals with high and low surgical rates, both in surgically and non-surgically treated patients (Fig. 3). At hospitals with a high surgical rate, the fractures were caused by high-energy trauma in 4.2% of the cases, low-energy trauma in 90.1%, and not specified in 5.7%. The corresponding figures for hospitals with low surgical rates were similar: 3.5%, 89.6%, and 6.9%. The injury mechanism in hospitals with a high surgical rate was falling accidents in 91.1% of cases, transport accidents in 5.3%, accidents due to external forces in 1.9%, and not specified in 1.7%. The corresponding figures for hospitals with a low surgical rate were: 91.4%, 5.0%, 2.1%, and 1.5% [18].

**Fig. 4:**
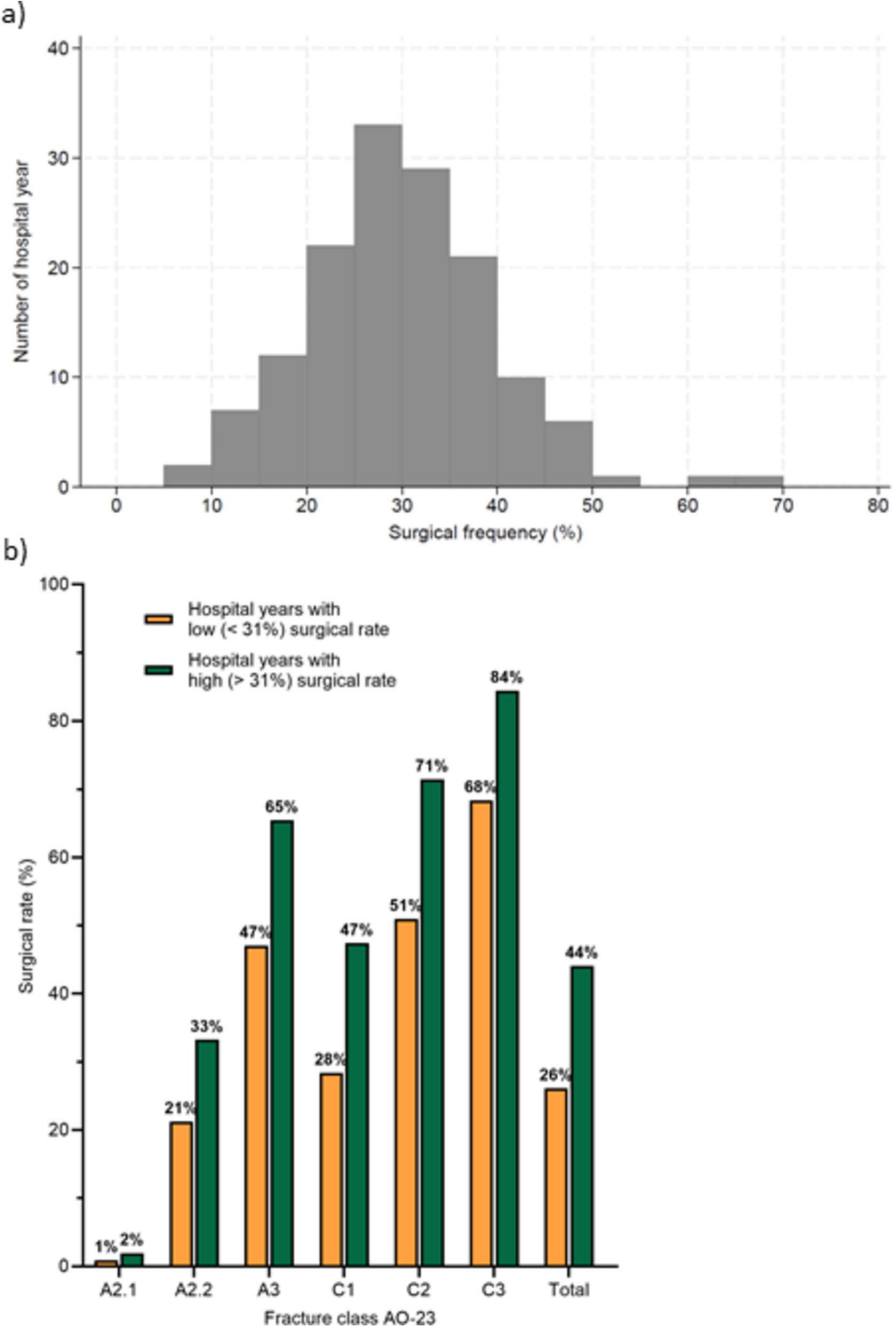
**a** The distribution of the number of hospital-years by surgical frequency for the treatment of distal radius fractures. **b** Surgical rates for AO-23 fracture classes dichotomized to lower (< 31%) or higher (> 31%) than the average surgical rate in the hospital year

The main instrumental variable was a continuous exposure to the surgical frequency of the fracture for each hospital-year, with a range between 7- 66% (Fig. 4a). The mean surgical rate for all patients was 31%. The tendency to perform surgery was strongly related to fracture severity. The average surgical rate per fracture class was 1% for A2.1, 28% for A2.2, 58% for A3, 40% for C1, 64% for C2, and 79% for C3.

In Fig. 4b, the surgical rates per fracture class have been dichotomized as HY below or above the mean surgical rate for all fractures. Consequently, the higher surgical rates observed at surgically active hospitals were evident across all fracture types.

The first-stage partial R^2^ in the two-stage least squares analysis was 0.033, indicating a modest association between the instrument and the exposure. The Kleibergen–Paap rk Wald F-statistic is 409, significantly higher than the Stock–Yogo critical value for a maximum 10% IV bias (16.38). This shows that the instrument is strong, making weak-instrument bias negligible.

**Fig. 5:**
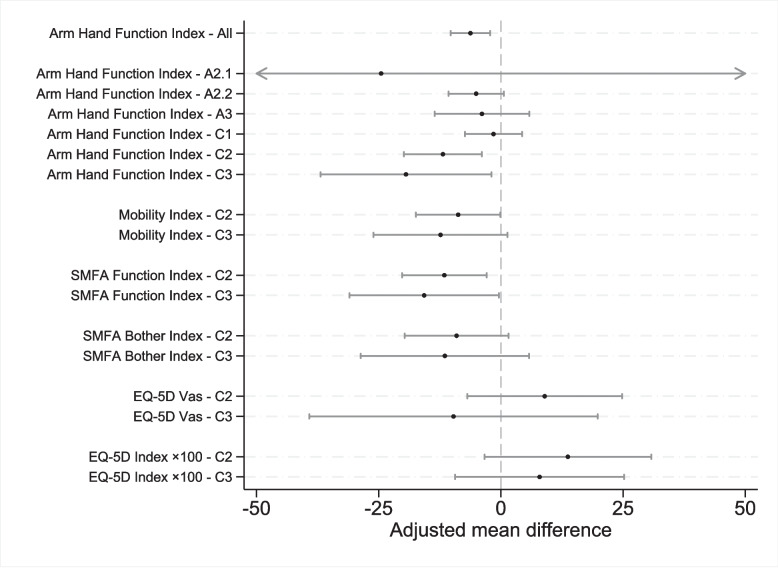
PROM adjusted mean differences between surgical and non-surgical treatments (95% CI). A negative value indicates a positive effect from surgical treatment for all scores, except for EQ5D. The Arm Hand Function Index is provided for all fracture classes. The remaining scores are shown only for 23-C2 and 23-C3, since no significant difference was observed in 23-A2.1-C1

Surgical treatment was, on average, associated with a 6.2 (95% CI 2.2–10.3; *p =* 0.002) units lower Arm and Hand Function Index than non-surgical treatment (Fig. 5). This difference is below the MCID for the Arm and Hand Function Index. However, the positive association favouring surgery increased with more severe fracture classes. We observed a clinically significant lower Arm and Hand Function Index after surgical treatment of intra-articular comminuted fractures. In fracture type 23-C2, the Arm and Hand Function Index was 11.9 (95% CI 3.9–19.8; *p =* 0.004) units lower, and in 23-C3, 19.4 (95% CI 1.9–36.9; *p =* 0.029) units lower than with non-surgical treatment. There was a moderate positive association with surgical treatment in dorsally angulated extraarticular fractures (23-A2.2), but the difference (5.1, 95% CI −0.59 – 10.7; *p =* 0.079) was below the MCID and not statistically significant. In other extra-articular fractures (23-A2.1 and 23-A3) and simple intra-articular fractures (23-C1), the benefits of surgical treatment were small and not statistically significant (Fig. 5). The potential benefit from surgery was greater for the Arm and Hand Function Index than for the Mobility Index.

Out of 13,356 fractures, 711 fractures (5%) were treated at two hospitals. A referral from one hospital to another can be caused by either a need for a higher level of competence or the patient returning to their home hospital. Seventy out of 711 fractures were classified as consultant patients.

A sensitivity analysis, excluding trauma level 1 hospitals (one-third of the cases), still showed a 6.1 (11.3–0.91; *p =* 0.021) lower Arm and Hand Function Index for surgery compared to non-surgical treatment. Additionally, instrumental variable analysis without confounding factors indicated an overall benefit from surgery on the Arm and Hand Function Index of 5.3 (95% CI 2.2–8.4; *p =* 0.001), and for the random effects generalized least squares regression without an instrumental variable, we found a benefit of 1.6 (0.9–2.2; *p <* 0.001).

**Fig. 6:**
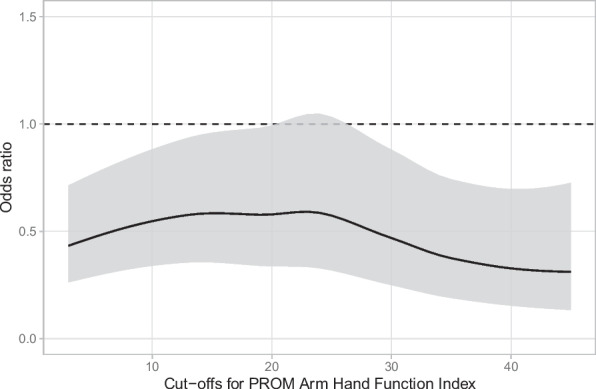
Odds ratios of surgery versus non-surgery from multiple instrumental variable logistic regression analyses, using dichotomized Arm Hand Function Index outcomes with cut-offs ranging from 3 to 45

Dichotomizing PROM at various levels (from 3 through 45, in one-unit steps) and analyzing it using instrumental variable logistic regression analyses (one for each cutoff) indicated a positive relationship between surgery and good subjective outcomes at all PROM cutoff levels (Fig. 6).

The findings remained consistent when (a) patients with associated injuries, (b) other distal radius fractures during the study period 2013–2018, (c) bilateral fractures, (d) surgically treated fractures after initial non-surgical treatment, and e) fractures sent to another hospital due to consultation were excluded (Supplementary Figure i).

The PROM response rate was 40% (*n =* 13,356 responses out of 33,768 fractures). The fracture class distribution was similar among non-responders, patients responding only to PROM at baseline, and patients responding to PROM at both baseline and follow-up (Supplementary Figure ii a). PROM response at baseline showed a similar distribution between those with a PROM response only and those with a PROM response at both baseline and follow-up (Supplementary figure ii b).

The complementary instrumental analysis of Delta PROM (the difference between PROM 1 and PROM 0) reduced the positive effect of surgery for all fractures except fracture class C3. A detailed analysis of Delta PROM distribution revealed a recurring error in some patients’ PROM 0 registrations (recall of the situation before the fracture event, with a response provided after the fracture) in SFR. This prompted us to rely solely on PROM 1 in our calculations (Supplementary Fig ii).

Secondary outcomes (SMFA Function Index, SMFA Bother Index, and EQ-5D Index) also reveal an association between surgical treatment and improved patient-reported outcomes in fracture class C2/C3. (Fig. 5). Only EQ-5D VAS showed no statistical difference between surgery and non-surgery.

In high-rate hospitals, the interventions were plate fixation in 89% of cases, external fixation in 2%, pin fixation in 7%, and other methods in 2%. In low-rate hospitals, the corresponding numbers were plate fixation in 75% of cases, external fixation in 6%, pin fixation in 15%, and other methods in 4%.

## Discussion

This is the first time an instrumental variable design has been used to evaluate the treatment effect of distal radius fractures. Using variation in surgical frequency across hospitals as a natural experiment, this study found that surgical treatment of 23-C2 and C3 fractures is associated with better PROM outcomes than non-surgical treatment. However, this does not mean that surgery is always the best option for these fracture types. In practice, our findings suggest that a higher tendency to treat 23-C2/C3 fractures surgically may be beneficial. The average surgical rate for HY with a low surgical rate for 23-C2 fractures in this study is 51%, and for HY with a high surgical rate, it is 71%. The corresponding rates for 23-C3 fractures are 68% and 84%. Within these ranges, a higher surgical rate generally indicated a more advantageous result. Our study did not support an increased tendency to treat other DRF fracture types surgically within the surgical rate ranges reported (Fig. 4).

Several moderately large randomized controlled trials in adults have assessed subjective and objective outcomes after surgical versus non-surgical treatment for distal radius fractures across different AO-23 fracture classes. Mulders et al. [19] studied well-reduced extra-articular distal radius fractures (23-A2/A3) and concluded that surgical treatment should be considered for this injury. We only observed a tendency toward better outcomes after extra-articular distal radius fractures and conclude that a higher surgical rate within the current rates for 23-A in our study (Fig. 4) is not advantageous. Similar studies on unstable intra-articular distal radius fractures (23-C) in adults support the use of surgical treatment for unstable intra-articular fractures, even in older patients, consistent with our findings [20–22].

Our results support the view that intra-articular involvement is a risk factor for inferior subjective results after non-surgical treatment of distal radius fractures. Intra-articular involvement as a risk factor for late fracture displacement has been studied several times over the past few decades, but the evidence has been conflicting. Lafontaine et al. [23] treated 112 distal radius fractures, of which 24 were intra-articular, with closed reduction and plaster cast fixation. They found a significant correlation between intra-articularity and fracture displacement. On the other hand, Wadsten et al. [24] examined 398 distal radius fractures treated non-surgically. Intra-articular involvement was seen in 117 fractures. They concluded that intra-articular involvement was not a risk factor for fracture displacement during treatment but did result in a reduced flexion–extension arc and a worse EQ-5D score. Late fracture displacement may be the reason for a poor outcome after non-surgical treatment in 23-C2/C3 fractures. However, given the conflicting evidence on fracture instability in previous studies, the poorer results in 23-C2/C3 may be due to factors other than malunion.

In our study, patients who underwent surgery after unsuccessful non-surgical treatment were included in the surgical group. As a result, PROM data in the non-surgical group were reported only by patients with fractures deemed suitable for non-surgical management, even after the initial follow-up, and not by patients with redisplaced fractures at 10 days. Our findings suggest that although a 23-C2/C3 fracture is often assessed as stable enough for non-surgical treatment, surgical intervention may still be associated with better subjective outcomes. This highlights the importance of thorough evaluation after closed reduction and at the first follow-up at 10 days to prevent overlooking surgical treatment in the most unstable fractures. We believe that simply increasing the surgical rate in 23-C2/3 fractures is not appropriate; instead, a more rigorous selection process for non-surgical treatment should be adopted. Meanwhile, the instrumental variable regression analysis of dichotomized PROM at various levels (Fig. 6) revealed a positive effect of surgical treatment across all PROM levels. This indicates that surgical intervention is associated with improved outcomes not only in the most severe cases. Surgical treatment may not only prevent disastrous outcomes in 23-C2/C3 but is also generally associated with better outcomes across multiple dimensions of fracture severity, for example, fracture displacement.

The main strength of our study is its use of a natural experiment, in which patients with similar characteristics were treated with either surgical or non-surgical methods, with the degree of surgical treatment varying by setting and year. This approach eliminates the need to fully account for selection processes and confounding factors. In the cohort, we show that hospitals with low and high surgical rates experience the same range of fractures each year (Fig. 3). When individual data on treatment type and PROM are available, only the surgical rate affects patient-reported outcomes. Another key strength is the large number of patients included, which improves statistical robustness. The SRF's provision of individual data on treatment and outcomes (PROM) is also valuable. Volar plate fixation remains the most common procedure across all hospitals. However, since long-term outcomes after plate fixation and percutaneous methods are similar, we believe that the positive effect observed in hospitals with high surgical rates is primarily attributable to the higher surgical rate itself rather than to the specific procedure. To reduce uncertainty in our results, we performed several sensitivity analyses, all of which supported our conclusion.

The study has several limitations. We assume that all hospitals encounter the same types of distal radius fractures each year, but this assumption has not been thoroughly verified. Out of 13,356 fractures, 70 fractures were referred to another hospital for consultation. Referrals due to a need for higher competence are most likely sent to hospitals with a high surgical rate. Therefore, we believe that this potential misregistration of some severe fractures at hospitals with high surgical rates may have slightly increased both the fracture severity and the surgical rates at these hospitals. However, in a sensitivity analysis, these fractures were excluded, and the main result remained unchanged (Supplementary figure i, panel e).

AO fracture class is most often assessed using conventional radiographs. Sometimes, CT is performed at the physician’s discretion. The Swedish Fracture Register contains no information about the type of radiological investigation performed. CT is often performed preoperatively and may reveal additional information about the fracture not seen on radiographs, such as undisplaced intra-articular engagement. This means that hospitals with high surgical rates may register more intra-articular fractures than those with low surgical rates. However, in clinical practice, we believe that the decision to treat a fracture surgically has already been made by the time the CT is ordered, and that performing a CT scan doesn’t increase the surgical rate. In addition, the distribution of fracture classes between HY with high or low surgical rates is similar.

A previous study validated the AO classification of distal radius fractures [25]. The accuracy of this classification for DRF was moderate, slightly lower than that for other fracture sites. Despite this, the distribution of fracture classes is nearly the same for surgically treated fractures in HY with high, compared to low, surgical rates, and for non-surgically treated fractures in HY with high, versus low, surgical rates (Fig. 3). We also reported that both the energy level and mechanism of injury are similar among hospitals with high and low surgical rates. However, it is possible that hospitals with high surgical rates treated more severe fractures, as measured by displacement and comminution. It is worth noting that we found an association between surgical treatment and better subjective outcomes in 23-C2/C3 fractures, despite potentially more severe fractures at hospitals with higher surgical rates. This suggests that the potential benefit of surgical treatment in 23- C2/C3 might be even greater if our initial assumption of equal fracture severity is not met.

The instrument should affect the outcome only through the treatment, but this assumption cannot be empirically verified. For example, hospitals with high surgical rates might have more skilled surgeons because of subspecialization at larger hospitals. Conversely, hospitals with low surgical rates might provide more effective non-surgical treatments. This potential bias was minimized by using surgical rates from HY rather than the average rates across all years at the hospitals. Since volar plate fixation is generally not considered a particularly demanding procedure for the surgeon, we do not believe that differences in surgical skill among hospitals account for the positive effects of surgical treatment at hospitals with high surgical rates; rather, the treatment choice itself is responsible. In a sensitivity analysis, trauma level 1 hospitals were excluded, and the results remained unchanged. This strengthens our confidence that treatment quality was similar across hospitals of different sizes. Additionally, the potential benefit of surgery was greater for the Arm and Hand Function Index than for the Mobility Index, reinforcing the idea that higher surgical rates are associated with better outcomes.

The PROM response rate was only 40%. There are two reasons for the non-response to the questionnaire. The first is administrative shortcomings at the registering hospital. The PROM 0 questionnaire is sent only to patients registered within 2 weeks of their injury, and PROM 1 is sent only to patients who respond to PROM 0. This non-response is most likely random with respect to the subjective outcome. The second reason is that patients fail to return the questionnaire. This may conceal a confounder, such as the patients’ review of the treatment. However, this has been examined in a study by Juto et al. [26]. They compared responders and non-responders with upper-extremity fractures from the Swedish Fracture Register by contacting non-responders by phone. They found that responders and non-responders did not differ in treatment satisfaction, and that only 1% of all non-responses were due to dissatisfaction. We found similar fracture severity among patients who didn´t respond to PROM, only responded to baseline PROM, or answered both baseline and follow-up PROM (Supplementary Figure ii a). We also found similar baseline PROM levels among patients responding only to baseline PROM compared with those responding to both baseline and follow-up PROM (Supplementary Figure ii b). All these findings indicate a low risk of selection bias in the study.

Another limitation of the study is that fracture displacement and other fracture properties, aside from AO-23 sub-classes, energy level, trauma mechanism, gender, and age, were not included. However, we believe there is no significant difference in fracture displacement or other properties among patient groups from different HYs. It is also well known that the correlation between radiographic parameters and subjective end result is poor. Several previous studies have shown that surgical treatment of distal radius fractures results in better radiographic outcomes but similar subjective outcomes compared with non-surgical treatment [21, 27]. Therefore, a comparison of subjective end results without radiographic parameters can stand alone.

The decision between surgery and non-surgical treatment depends not only on fracture classification but also on the degree of displacement. RCTs on DRF comparing surgical and non-surgical treatments typically include only unstable fractures, aiming to identify the best treatment for all fracture types studied. Our register-based study includes all fractures, from undisplaced to severely displaced, meaning it also covers fractures across all classes that can undoubtedly be treated without surgery. Therefore, the positive effect of surgery observed in 23-C2/C3 fractures should not be generalized to all such fractures but should be considered one of many factors when deciding between surgical and non-surgical options. It also serves as a guide for setting optimal surgical rates for 23-C2/C3 fractures. Indicated by our results, we suggest that only a moderate proportion of 23-C2 fractures, and even a smaller proportion of 23-C3 fractures, should be treated without surgery.

Our pre-planned goal was to use Delta PROM, which measures the difference between PROM 1 (at 1 year) and PROM 0 (pre-injury, as recalled). However, many patients reported a significantly higher PROM 0 than PROM 1 (Supplementary Figure iii b). Since it is impossible to have better health one year after a distal radius fracture than pre-injury levels, we suspected these patients misunderstood the instructions and reported their post-injury health shortly after the fracture. Several previous studies have shown that recalled pre-injury patient-reported outcomes often exceed population norms [28]. In general, older patients and women perform slightly worse on baseline function scales [29]. We also found that estimating pre-injury PROM after the injury was unreliable. We observed an even distribution of age and gender between HY groups with high and low surgical rates. It is likely that there is no significant difference in pre-injury status across HY groups. Instead of using potentially misleading PROM 0 values, we therefore relied only on PROM 1 as the primary outcome.

The SFR uses EuroQol Group 5-Dimension (EQ5D) and the Short Musculoskeletal Function Assessment (SMFA), both of which are considered generic PROMs designed to measure changes in overall aspects of a patient's health. There is conflicting evidence regarding the responsiveness of generic PROMs such as EQ5D and SMFA in distal radius fractures compared with region-specific DASH or wrist-specific PRWE. Nevertheless, both EQ5D and SMFA, especially the Arm and Hand Function Index from SMFA, were sufficiently sensitive in our study.

## Conclusion

On average, surgical treatment of distal radius fractures 23-C2 and 23-C3 is associated with better patient-reported outcomes than non-surgical treatment.

## Supplementary Information

Supplementary Material 1: Supplementary Figure i. The Arm Hand Function Index adjusted mean difference between surgical and non-surgical treatment (95% CI) after excluding: a) patients with associated injuries from 1 year before to 1 year after the index fracture (*n=*1460 patients), b) patients with another distal radius fracture during the study period 2013-2018 (*n=*602 fractures in 384 patients), c) patients with bilateral fractures at the index fracture (*n=*214 fractures in 107 patients), d) surgically treated fractures after initial non-surgical treatment (*n=*1118) or e) fractures sent to another hospital due to consultation (*n=*70). A negative value indicates a positive effect from surgical treatment for all scores. These sensitivity analyses confirm the positive effects of surgery in fracture classes AO 23- C2/C3.

Supplementary Material 2: Supplementary Figure ii. a) Fracture severity distribution by number of PROM questionnaire responses. b) Distribution of baseline PROM responses in those with a PROM response only at baseline (left panel) and in those who responded to the PROM questionnaire both at baseline and follow-up (right panel).

Supplementary Material 3: Supplementary Figure iii. a) The delta Arm Hand Function Index adjusted mean difference (difference between PROM 1 and PROM 0) between surgical and non-surgical treatments (95% CI). A negative value indicates a positive effect from surgical treatment. The previously observed positive effect of surgery only remains in fracture class C3. b) A histogram of delta Arm Hand Function Index (difference between PROM 1 and PROM 0). The most common result was no (zero) difference between PROM 1 and 0 (dark grey thin bar in the diagram). A negative value indicates less disability at 1 year than before the fracture. Since this is unlikely, it probably results from a recurrent misunderstanding among patients. Instead of recalling their pre-injury PROM, many patients reported their current difficulties shortly after the fracture. This misunderstanding hampers accurate assessment of the benefits of surgery. This is why we used only PROM 1 in this study, despite Delta PROM being more methodologically accurate.

## Data Availability

The dataset used and analysed in this study is available from the corresponding author on reasonable request.
