# Supplementary figures and images for: Surgical versus non-surgical treatment of intra-articular comminuted distal radius fractures (AO 23-C2/C3) is associated with better patient-reported outcomes: an instrumental variable analysis using a national Swedish cohort
Source: BMC Musculoskelet Disord. 2026 May 4;27:386. doi: 10.1186/s12891-026-09900-z (PMC13147605; doi:10.1186/s12891-026-09900-z)

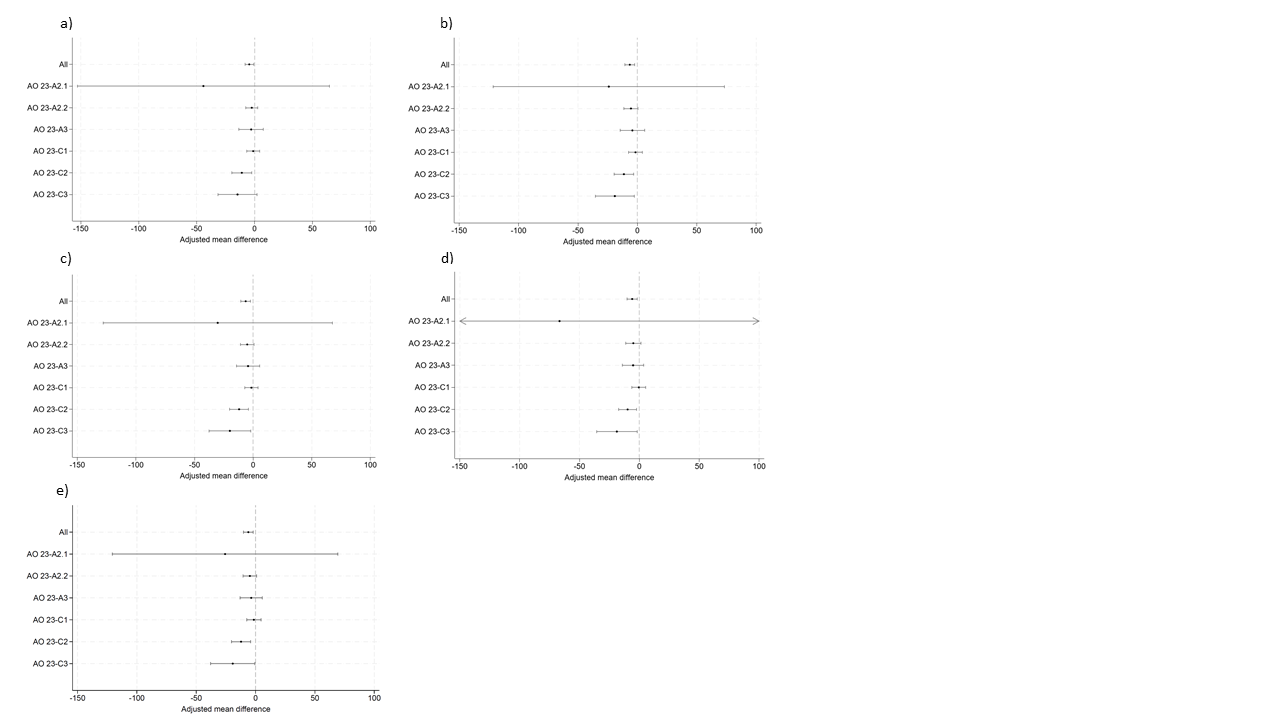

Supplement: Supplementary file 1 — Supplementary Material 1: Supplementary Figure i. The Arm Hand Function Index adjusted mean difference between surgical and non-surgical treatment (95% CI) after excluding: a) patients with associated injuries from 1 year before to 1 year after the index fracture (n=1460 patients), b) patients with another distal radius fracture during the study period 2013-2018 (n=602 fractures in 384 patients), c) patients with bilateral fractures at the index fracture (n=214 fractures in 107 patients), d) surgically treated fractures after initial non-surgical treatment (n=1118) or e) fractures sent to another hospital due to consultation (n=70). A negative value indicates a positive effect from surgical treatment for all scores. These sensitivity analyses confirm the positive effects of surgery in fracture classes AO 23- C2/C3. [file 12891_2026_9900_MOESM1_ESM.png]

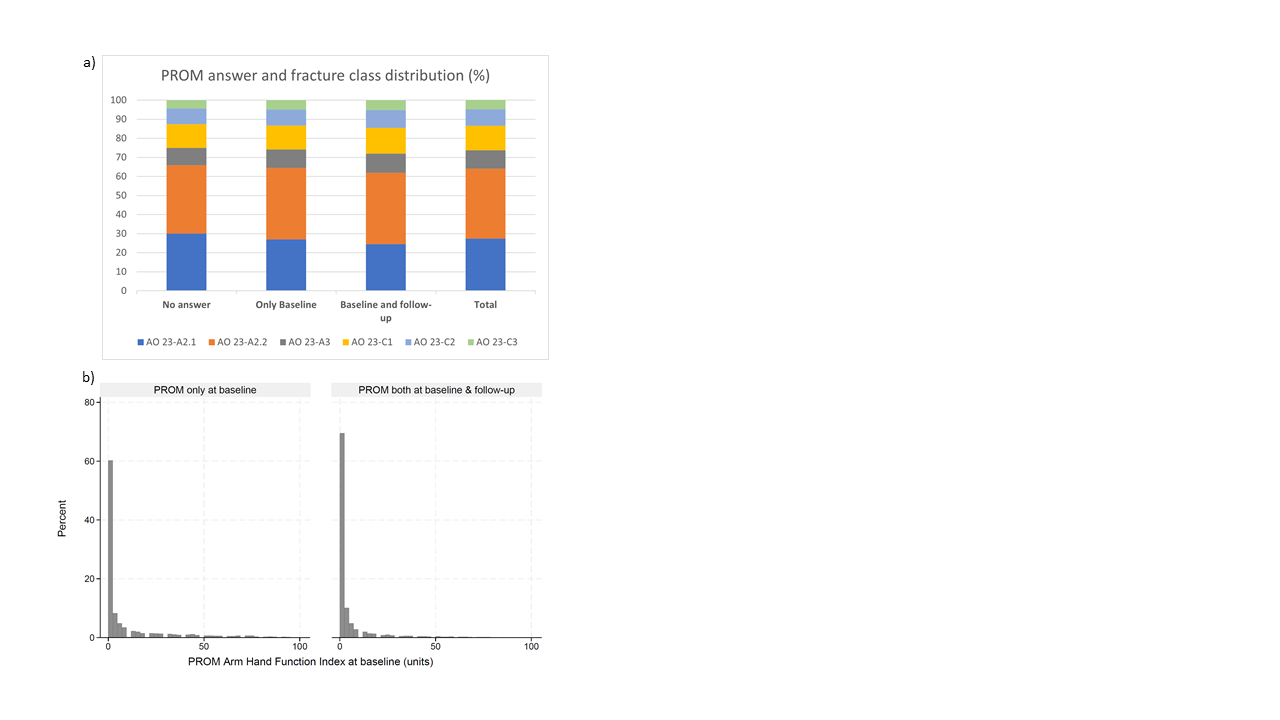

Supplement: Supplementary file 2 — Supplementary Material 2: Supplementary Figure ii. a) Fracture severity distribution by number of PROM questionnaire responses. b) Distribution of baseline PROM responses in those with a PROM response only at baseline (left panel) and in those who responded to the PROM questionnaire both at baseline and follow-up (right panel). [file 12891_2026_9900_MOESM2_ESM.png]

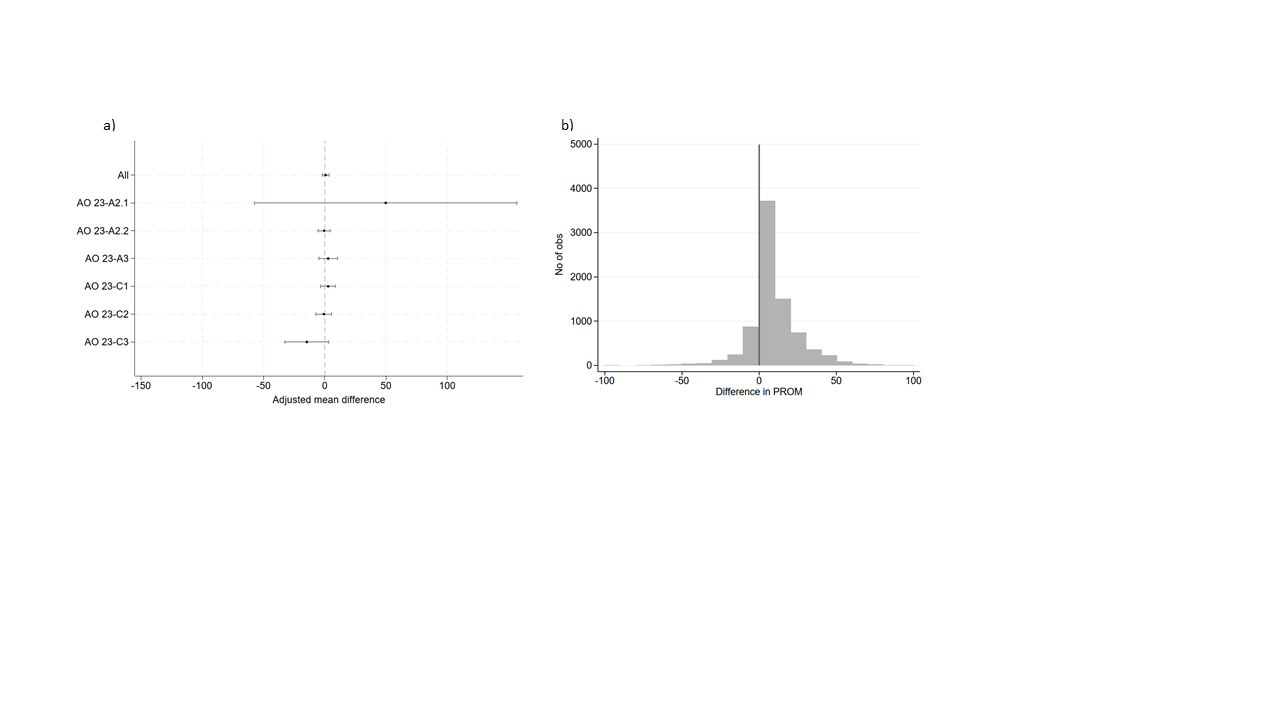

Supplement: Supplementary file 3 — Supplementary Material 3: Supplementary Figure iii. a) The delta Arm Hand Function Index adjusted mean difference (difference between PROM 1 and PROM 0) between surgical and non-surgical treatments (95% CI). A negative value indicates a positive effect from surgical treatment. The previously observed positive effect of surgery only remains in fracture class C3. b) A histogram of delta Arm Hand Function Index (difference between PROM 1 and PROM 0). The most common result was no (zero) difference between PROM 1 and 0 (dark grey thin bar in the diagram). A negative value indicates less disability at 1 year than before the fracture. Since this is unlikely, it probably results from a recurrent misunderstanding among patients. Instead of recalling their pre-injury PROM, many patients reported their current difficulties shortly after the fracture. This misunderstanding hampers accurate assessment of the benefits of surgery. This is why we used only PROM 1 in this study, despite Delta PROM being more methodologically accurate. [file 12891_2026_9900_MOESM3_ESM.png]
